# Complex high-risk indicated PCI (CHIP-PCI): is it safe to let fellows-in-training perform it as primary operators?

**DOI:** 10.1136/openhrt-2024-003131

**Published:** 2025-01-30

**Authors:** Majd B Protty, Saad Hasan, Diluka Premawardhana, Mohammed Shugaa Addin, Holly Morgan, Shantu Bundhoo, Hussain Hussain, Zia Ul-Haq, Alexander Chase, David Hildick-Smith, Anirban Choudhury, Tim Kinnaird, Ahmed Hailan

**Affiliations:** 1 Sir Geraint Evans Cardiovascular Research Building, Cardiff University, Cardiff, South Glamorgan, UK; 2 Morriston Cardiac Centre, Swansea, UK; 3 University Hospital of Wales, Cardiff, Cardiff, UK; 4 King's College London, London, UK; 5 Grange University Hospital, Cwmbran, UK; 6 Sussex Cardiac Centre, Brighton and Sussex University Hospitals NHS Trust, Brighton, UK

**Keywords:** Percutaneous Coronary Intervention, Inservice Training, Clinical Competence

## Abstract

**Background:**

Training in complex high-risk indicated percutaneous coronary intervention (CHIP-PCI) has frequently been reserved for established operators (consultants/attending) with trainees (fellows-in-training or FIT) being often discouraged from carrying out such procedures as a primary operator due to their high-risk nature. Whether the outcomes of these cases differ if the primary operator is a supervised FIT compared with a consultant is unknown.

**Methods:**

Using multicentre PCI data from three cardiac centres in South Wales, UK (2018–2022), we identified 2295 CHIP-PCI cases with a UK-BCIS CHIP Score of 3 or more. These were then divided by primary operator status (supervised FIT vs consultant); the primary outcome was in-hospital major adverse cardiac events (IH-MACCE). Multivariate logistic models were developed to adjust for differences in baseline and procedural characteristics.

**Results:**

The primary operator in 838 (36%) of the PCIs was a supervised FIT. Baseline and procedural characteristics had lower complexity in CHIP-PCI cases carried out by supervised FIT vs consultant. In a multivariate-adjusted model, supervised FIT procedures were associated with lower odds of concurrent valve disease (OR 0.45, 95% CI: 0.29 to 0.69), dual access (OR 0.58, 95% CI: 0.41 to 0.83), cutting/scoring balloons (OR 0.59, 95% CI: 0.44 to 0.79) and rotational atherectomy (OR 0.60, 95% CI: 0.42 to 0.87). After adjusting for all variables, however, there was no difference in the primary outcome (OR 0.72, 95% 0.34 to 1.51) or any secondary outcomes. Sensitivity analyses restricted to patients with higher CHIP Scores (4+ and 5+) showed comparable IH-MACCE.

**Conclusions:**

Training FIT as primary operators in CHIP-PCI appears to be feasible and safe and can be delivered within the standard training programme. The comparable outcomes are likely driven by the two-operator ‘buddy’ effect that a FIT supervised by a consultant benefits from.

**WHAT IS ALREADY KNOWN ON THIS TOPIC**

- It is unknown whether the outcomes of complex high-risk indicated percutaneous coronary intervention (CHIP-PCI) vary when the primary operator is a fellow-in-training (FIT) or an established consultant.

**WHAT THIS STUDY ADDS**

- Consultant-led cases were higher risk than FIT-led cases, but after adjusting for all clinical and procedural characteristics, there was no difference in the primary outcome of in-hospital major adverse cardiac events (OR 0.72, 95% 0.34 to 1.51) or any other procedural complications.

**HOW THIS STUDY MIGHT AFFECT RESEARCH, PRACTICE OR POLICY**

- Training FIT as primary operators in CHIP-PCI appears to be feasible and safe and can be delivered within the standard training programme.

## Introduction

 Complex high-risk indicated percutaneous coronary intervention (CHIP-PCI) requires advanced techniques such as left main stenting, calcium modification and bifurcation stenting, traditionally managed by highly experienced operators (consultants/staff or attending interventional cardiologists) due to their high-risk nature.^1 2^ Integrating interventional cardiology fellows-in-training (FIT, specialist registrars, senior registrars) into CHIP-PCI involves balancing rigorous training with patient safety, raising important questions about the appropriateness for FIT to perform these procedures as primary operators under supervision.^3^

The uncertainty regarding whether supervised FIT, as primary operators for CHIP-PCI, can achieve comparable and safe outcomes is crucial, particularly, as the complexity of coronary artery disease continues to increase^4 5^ and the need for FIT to be prepared for independent CHIP-PCI practice becomes more pressing. This challenge underscores the importance of developing more structured and effective training programmes to ensure FIT are adequately equipped for future practice.^3^

This paper explores whether it is safe for supervised FIT to act as primary operators in CHIP procedures and whether their outcomes differ from those of consultant primary operators. As the demand for CHIP-PCI rises, understanding these dynamics is vital for ensuring patient safety and enhancing the effectiveness of training programmes, given the current lack of decisive evidence in the literature.^4^

## Methods

### Study participants

We conducted a retrospective analysis of PCI registries from three cardiac centres in South Wales, UK—Morriston Hospital in Swansea, University Hospital of Wales in Cardiff and the Grange University Hospital in Cwmbran—covering the period from January 2018 to December 2022. The retrospective anonymised study design was approved by the local clinical governance board.

The local PCI registries conform to the guidelines from the National Institute of Cardiovascular Outcomes Research (NICOR) and the British Cardiac Intervention Society (BCIS) to inform patients and the public on PCI outcomes in UK National Health Service (NHS) centres.^6^ UK interventional cardiologists are mandated to enter all PCI procedures undertaken, including complications, onto these datasets as part of professional revalidation. In addition, data on primary operator status is routinely captured as part of this local database for every PCI procedure completed, which facilitates splitting the data into two groups (FIT and consultant) for comparison.

### Design of the study

The following clinical parameters in all patients undergoing PCI were documented and stratified by operator status (FIT/consultant): age, gender, height, weight, acute coronary syndrome (ACS) status, New York Heart Association (NYHA) heart failure class, Canadian Cardiovascular Society (CCS) angina class, diabetes mellitus, left ventricular ejection fraction, smoking history (current or ex-smoker), hypertension, hypercholesterolaemia, previous myocardial infarction, previous coronary artery bypass grafting (CABG), family history of coronary artery disease (CAD), previous PCI to any vessel, peripheral vascular disease, valvular heart disease, previous stroke, pre-procedural renal disease (defined by BCIS as creatinine >200 µmol/L, renal transplant history or dialysis) and primary operator status (consultant/FIT).

The following procedural variables were documented: left main PCI, 3-vessel PCI, graft PCI, intracoronary imaging (intravascular ultrasound, IVUS, or optical coherence tomography, OCT), use of pressure wire, femoral access, dual access, lesion length >60 mm, use of microcatheter, cutting balloon, rotational atherectomy, intravascular lithotripsy (IVL) and mechanical circulatory support. The primary outcome was in-hospital major adverse cardiac and cerebrovascular events, IH-MACCE, a composite of death, stroke and periprocedural infarct. Secondary outcomes included in-hospital death, as well as procedural complications (side branch loss, dissection, shock induced by procedure, perforation, slow flow and DC cardioversion (DCCV)).

CHIP-PCI was defined as cases with a UK-BCIS CHIP Score of 3 or higher, a threshold previously demonstrated to correspond with the top 20% highest risk for in-hospital MACCE.^5^ A sensitivity analysis for higher risk cases, defined by CHIP Scores 4+ or 5+, was also carried out to examine for interactions at higher risk groups. Patients requiring emergency revascularisation, such as those with ST-elevation myocardial infarction or cardiogenic shock, were excluded from the study, as were procedures performed out of hours. Patients and the public were not involved in the design of this study.

### Statistical analyses

Statistical analysis was completed using the R coding environment (Open Source). Multiple imputations were carried out using the *mice* package to reduce the potential bias from missing data (online supplemental table S1), assuming missingness completely at random mechanisms. Chained equations were used to impute data for variables with missing information and generated five datasets to be used in the analyses. The dataset was split by primary operator status (supervised FIT vs consultant) to examine differences in hospital outcomes including IH-MACCE. Crude differences in baseline variables were explored using a χ² test for categorical variables and the Wilcoxon-Mann-Whitney test for continuous variables. Multivariate logistic regression models adjusted for baseline differences were used to identify covariates independently associated with in-hospital outcomes.

## Results

### Baseline characteristics of chip-PCI cases included in the study

The total number of CHIP-PCI cases identified during the study period was 2295 (online supplemental figure S1). The baseline clinical characteristics of the cohort are presented in table 1.

**Table 1:** Baseline characteristics of participants undergoing CHIP-PCI by consultant/trainee status

	Consultant	FIT	P value	All
N	1457	838		2295
Age (SD)	77.22 (10.77)	79.78 (9.72)	<0.001	78.16 (10.47)
CHIP Score (SD)	3.96 (1.32)	3.71 (1.10)	<0.001	3.87 (1.25)
CHIP Score 4+, n (%)	734 (50.4)	337 (40.2)	<0.001	1071 (46.7)
CHIP Score 5+, n (%)	359 (24.6)	164 (19.6)	0.006	523 (22.8)
BMI (SD)	27.56 (5.32)	27.30 (5.12)	0.276	27.46 (5.25)
Age >80, n (%)	834 (57.2)	591 (70.5)	<0.001	1425 (62.1)
Female, n (%)	739 (51.1)	490 (58.7)	0.001	1229 (53.9)
ACS, n (%)	934 (64.1)	641 (76.5)	<0.001	1575 (68.6)
CCS class 3+, n (%)	489 (61.4)	190 (56.9)	0.175	679 (60.1)
NYHA class 3+, n (%)	84 (30.8)	46 (37.1)	0.259	130 (32.7)
Diabetes, n (%)	483 (33.8)	246 (29.6)	0.045	729 (32.3)
EF <30%, n (%)	115 (8.1)	50 (6.2)	0.119	165 (7.4)
Smoking history, n (%)	392 (61.6)	236 (61.1)	0.927	628 (61.4)
FH of CAD, n (%)	798 (59.4)	377 (48.9)	<0.001	1175 (55.6)
Hypertension, n (%)	1064 (73.0)	630 (75.2)	0.280	1694 (73.8)
Hypercholesterolaemia, n (%)	828 (56.8)	557 (66.5)	<0.001	1385 (60.3)
Previous MI, n (%)	337 (23.1)	196 (23.4)	0.928	533 (23.2)
Previous CABG, n (%)	84 (5.8)	53 (6.3)	0.651	137 (6.0)
Previous PCI, n (%)	306 (21.0)	181 (21.6)	0.777	487 (21.2)
PVD, n (%)	268 (18.4)	158 (18.9)	0.828	426 (18.6)
Valve disease, n (%)	114 (7.8)	32 (3.8)	<0.001	146 (6.4)
Stroke, n (%)	170 (11.7)	85 (10.1)	0.294	255 (11.1)
Renal disease, n (%)	174 (12.5)	77 (9.4)	0.033	251 (11.4)

ACSacute coronary syndromeBMIBody Mass IndexCABGcoronary artery bypass graftCADcoronary artery diseaseCCSCanadian Cardiovascular Society ScoreCHIP-PCIcomplex high-risk indicated percutaneous coronary interventionEFejection fractionFHfamily historyFITfellow-in-trainingMImyocardial infarctionNYHANew York Heart Association ScorePVDperipheral vascular disease

Cases performed by FIT as primary operator had a lower average CHIP Score (3.71±1.10 vs 3.96±1.32, p<0.05), higher age (79.78±9.72 vs 77.22±10.77, p<0.05) and were more likely to be female (58.7% vs 51.1%, p<0.05), have presented with ACS (76.5% vs 64.1%, p<0.05) and have hypercholesterolaemia (66.5% vs 56.8%, p<0.05) (table 1). FIT cases had fewer patients with valvular heart disease (3.8% vs 7.8%, p<0.05), diabetes (29.6% vs 33.8%, p<0.05) and renal disease (9.4% vs 12.%, p<0.05).

### Procedural characteristics of chip-PCI cases

Supervised FIT cases had a lower proportion of left main stem PCI (21.4% vs 32.7%, p<0.05), 3-vessel PCI (19.5% vs 27.4%, p<0.05), dual access (8.2% vs 18.9%, p<0.05) and mechanical circulatory support (2.1% vs 5.1%, p<0.05) (table 2). There was also a lower proportion of cases using intracoronary imaging, femoral access, microcatheters, cutting/scoring balloons, IVL or rotational atherectomy (table 2). Overall, these suggest a lower procedural complexity in cases performed by FIT as primary operator.

**Table 2:** Procedural characteristics of participants undergoing CHIP-PCI by consultant/trainee status

	Consultant	FIT	P value	All
N	1457	838		2295
LMS PCI, n (%)	477 (32.7)	179 (21.4)	<0.001	656 (28.6)
3-vessel PCI, n (%)	399 (27.4)	163 (19.5)	<0.001	562 (24.5)
Graft PCI, n (%)	46 (3.2)	41 (4.9)	0.047	87 (3.8)
Intracoronary imaging, n (%)	664 (45.6)	242 (28.9)	<0.001	906 (39.5)
Pressure wire, n (%)	112 (7.8)	94 (11.2)	0.007	206 (9.0)
Femoral access, n (%)	289 (19.8)	98 (11.7)	<0.001	387 (16.9)
Dual access, n (%)	276 (18.9)	69 (8.2)	<0.001	345 (15.0)
Length >60 mm, n (%)	210 (14.4)	77 (9.2)	<0.001	287 (12.5)
Microcatheter, n (%)	183 (12.8)	57 (6.8)	<0.001	240 (10.6)
Cutting/scoring, n (%)	290 (20.2)	81 (9.7)	<0.001	371 (16.4)
Rotational atherectomy, n (%)	196 (13.7)	50 (6.0)	<0.001	246 (10.8)
IVL, n (%)	76 (5.3)	26 (3.1)	0.020	102 (4.5)
Mechanical LV support, n (%)	75 (5.1)	18 (2.1)	0.001	93 (4.1)

CHIP-PCIcomplex high-risk indicated percutaneous coronary interventionFITfellow-in-trainingIVLintravascular lithotripsyLMSleft main stemLVleft ventricular

**Figure 1:**
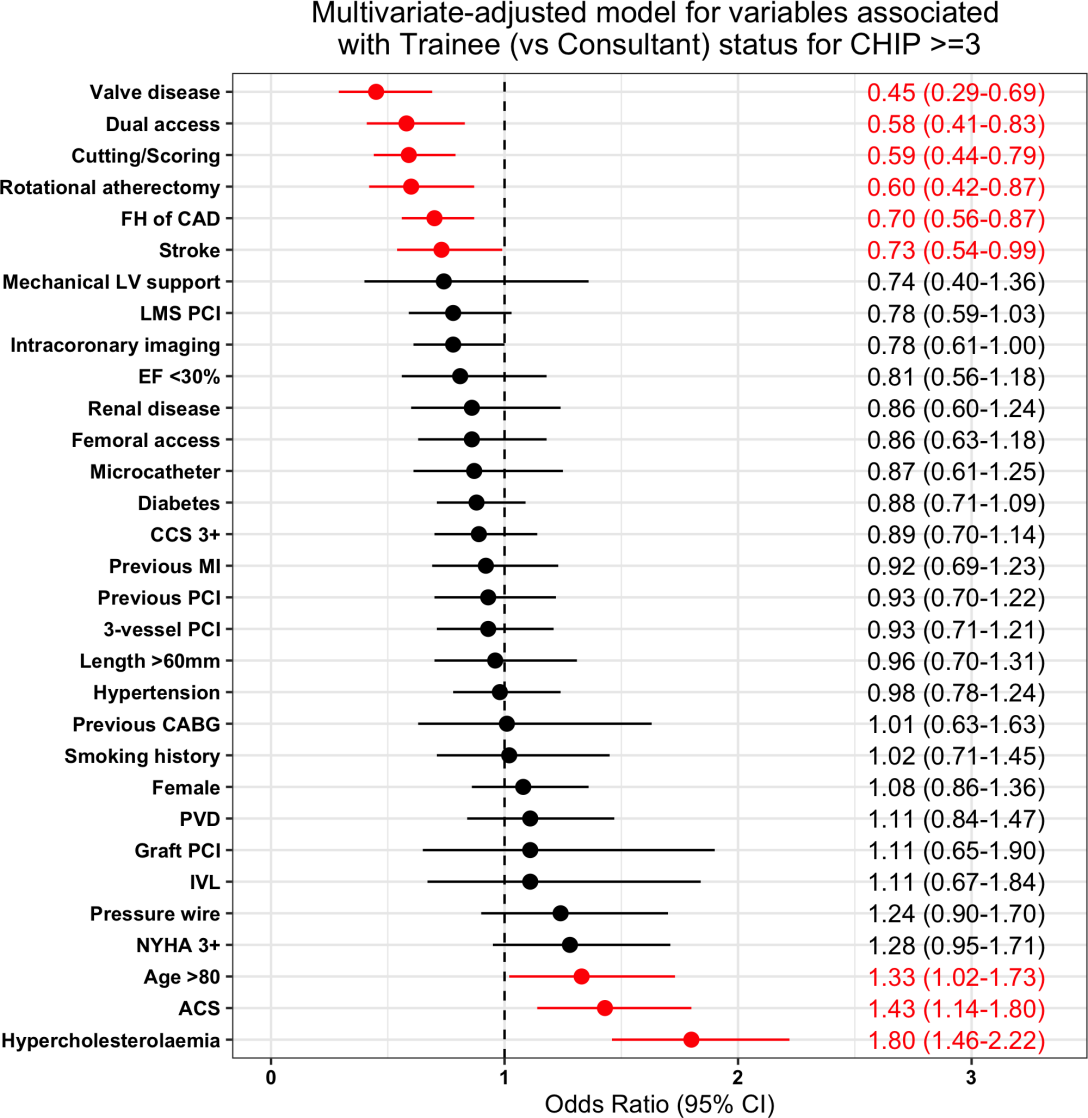
Multivariate-adjusted model for variables associated with FIT-led cases compared with consultant-led cases. The higher the OR, the more likely that the case is associated with FIT. FIT, fellow-in-training.

A multivariate logistic regression model was developed to better delineate variables associated with supervised FIT as primary operators compared with consultants (figure 1). Covariates more likely to be independently associated with FIT cases were age >80 years old (OR 1.33, 95% CI: 1.02 to 1.73), ACS presentation (OR 1.43, 95% CI: 1.14 to 1.80) and hypercholesterolaemia (OR 1.80, 95% CI: 1.45 to 2.22). Covariates less likely to be associated with FIT cases were valve disease (OR 0.45, 95% CI: 0.29 to 0.69), dual access (OR 0.58, 95% CI: 0.41 to 0.83), cutting/scoring balloons (OR 0.59, 95% CI: 0.44 to 0.79), rotational atherectomy (OR 0.60, 95% CI: 0.42 to 0.87), family history of coronary disease (OR 0.70, 95% CI: 0.56 to 0.87) and previous history of stroke (OR 0.73, 95% CI: 0.54 to 0.99).

### Clinical outcomes of chip-PCI by primary operator status

The unadjusted outcomes of cases included in this analysis are shown in table 3. These demonstrate that cases performed by supervised FIT have similar outcomes to consultant-led cases, except for coronary perforation which had a lower incidence in FIT-led cases (0.2% vs 1.2%, p<0.05).

**Table 3:** Unadjusted outcomes of CHIP-PCI by consultant/trainee status

	Consultant	FIT	P value	All
N	1457	838		2295
Tamponade, n (%)	0 (0.0)	1 (0.1)	0.779	1 (0.0)
Dissection, n (%)	19 (1.3)	5 (0.6)	0.164	24 (1.0)
Shock induction, n (%)	18 (1.2)	4 (0.5)	0.116	22 (1.0)
Perforation, n (%)	17 (1.2)	2 (0.2)	0.034	19 (0.8)
Slowflow, n (%)	21 (1.4)	13 (1.6)	0.976	34 (1.5)
DC cardioversion, n (%)	4 (0.3)	3 (0.4)	1.000	7 (0.3)
Sidebranch loss, n (%)	13 (0.9)	3 (0.4)	0.222	16 (0.7)
Stroke, n (%)	1 (0.1)	0 (0.0)	0.779	1 (0.0)
Periprocedural infarcts, n (%)	1 (0.1)	0 (0.0)	0.779	1 (0.0)
In-hospital death, n (%)	25 (1.7)	12 (1.4)	0.728	37 (1.6)
In-hospital MACCE, n (%)	25 (1.7)	12 (1.4)	0.728	37 (1.6)

CHIP-PCIcomplex high-risk indicated percutaneous coronary interventionDCdirect currentFITfellow-in-trainingMACCEmajor adverse cardiac and cerebrovascular events

**Figure 2:**
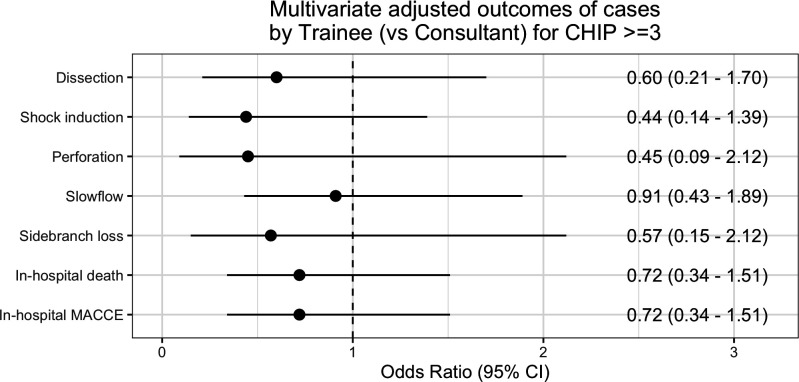
Multivariate-adjusted in-hospital outcomes of cases performed by FIT compared with consultant-led cases. This demonstrates no difference in outcomes between cases where primary operator is a supervised FIT or a consultant. FIT, fellow-in-training.

After adjusting for baseline and procedural covariates, there were no significant differences in in-hospital outcomes in cases performed by a supervised FIT compared with consultant as primary operator, including in-hospital MACCE and mortality (figure 2).

**Figure 3:**
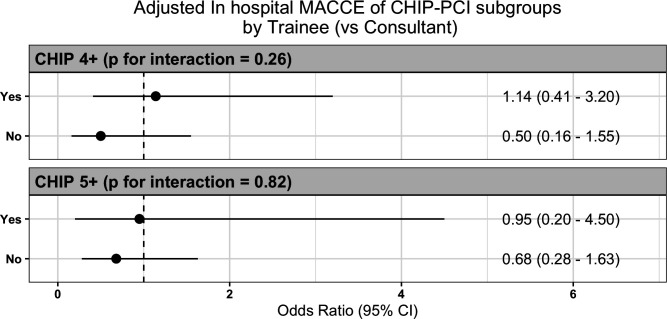
Sensitivity analysis of in-hospital MACCE comparing cases where primary operator is a supervised FIT or a consultant in subgroups with more complexity as defined by higher CHIP Scores. FIT, fellow-in-training; MACCE, major adverse cardiac and cerebrovascular events.

A sensitivity analysis for higher risk cases, defined by CHIP Scores 4+ or 5+, was performed (figure 3). This again demonstrates no difference in in-hospital MACCE between cases performed by a supervised FIT compared with consultant as primary operator.

## Discussion

In this multicentre study, we have demonstrated that training FIT as primary operators in CHIP-PCI is both feasible and safe. This can be effectively integrated into the standard training curriculum. While some trainees may benefit from supplementary training or additional fellowship opportunities,^7^ the findings indicate that FIT, when enrolled in the structured UK training programme, can achieve safe and successful CHIP-PCI outcomes under supervision. This structured approach not only equips FIT with essential technical skills but also builds their confidence in managing complex cases, a key component of their progression into independent operators.

Previous studies have shown that involving FIT in all-comer angiography and PCI cases does not lead to worse outcomes or more complications.^8^,^10^ However, these studies examined a predominantly lower risk cases in relatively younger patients; whereas, our study focuses on higher risk PCI cases defined by the recently described CHIP Score of 3 or more.^5^ The comparable outcomes between FIT and experienced consultants may be attributable to the ‘buddy’ system, wherein a FIT operates under close consultant supervision. This two-operator model facilitates real-time guidance, immediate correction of technical errors and collaborative decision-making, all contributing to the procedure’s safety and effectiveness. Moreover, it reduces the trainee’s learning curve, as observed in both coronary intervention and other procedural specialities.^6 11^ The consultant’s presence and supervision ensure patient safety while providing FIT with invaluable hands-on experience and mentorship. This dual-operator dynamic is instrumental in bridging the gap between theoretical learning and practical expertise, ultimately producing cardiologists capable of independently managing complex interventions.

FIT-led cases involved fewer enabling strategies and devices compared with consultant-led cases, suggesting that FIT were tasked with less technically challenging cases (table 2). This trend persisted even after adjusting for baseline comorbidities and overall procedural characteristics (figure 1). This may reflect case selection by supervising consultants, who assigned fewer procedures with rotational atherectomy and dual access (eg, chronic total occlusion) to FIT. This selection bias should be considered when interpreting these results, as it highlights the potential confounding (but likely appropriate) effect of case selection. Nonetheless, a significant proportion of FIT-led cases employed advanced tools without adversely impacting adjusted outcomes (table 2, figure 3).

Cases led by FIT demonstrated a lower utilisation of intracoronary imaging compared with consultant-led cases (table 2). This finding was unexpected, as intracoronary imaging provides an excellent educational opportunity for FIT, regardless of the procedural complexity. Several factors may explain this observation. First, supervisors may prioritise teaching fundamental PCI techniques over advanced imaging to simplify the learning process and accommodate the FIT’s position along the learning curve. Second, the additional time and complexity introduced by intracoronary imaging might lead supervisors to adopt a more streamlined approach during FIT-led procedures to maintain procedural efficiency.^12^ Lastly, resource allocation may influence decision-making, with advanced imaging reserved for cases where it is expected to offer substantial clinical benefit.^12^ Importantly, after adjusting for all baseline covariates, the difference in intracoronary imaging use between groups was no longer statistically significant (figure 1), suggesting that the complexity of baseline patient and procedural characteristics largely account for this finding.

The position of FIT on the learning curve significantly affects procedural outcomes, with greater experience and higher case volumes associated with better clinical results and better FIT performance.^13^,^16^ However, the rotational nature of the UK training scheme, where FITs rotate annually, limits the ability to assess each trainee’s operator volume or stage of skill development.^17^ This structure makes tracking procedural experience challenging in this study. It is likely however that trainers tend to assign more complex cases to experienced FITs, while novice trainees handle less demanding cases under closer supervision. This ‘case selection’ approach may influence FIT outcomes, a factor that the current study cannot directly address due to the rotational training model.

The sensitivity analysis in figure 3 offers further reassurance that supervised FIT cases remain safe and produce comparable outcomes, even as case complexity, measured by the CHIP Score, increases. However, due to limited sample sizes, cases with CHIP Scores above 5 could not be extensively studied in this dataset.

### Limitations of the study

Despite efforts to adjust for baseline differences, unmeasured confounders such as frailty, technical challenges or operator expertise may have influenced the results, a common limitation in retrospective studies. While a prospective randomised trial could address these issues, it may not be appropriate given the focus on patient safety. Additionally, although we used multiple imputation for missing data, the underlying statistical assumptions are difficult to verify. Furthermore, the BCIS database records primary/secondary operator status as FIT or consultant but does not specify the level/style of supervision (verbal vs practical) or hands-on assistance required (whether that is a consultant providing hands-on supervision or a FIT providing hands-on assistance) nor does it capture anatomical details like lesion complexity or bifurcation disease or concomitant procedures such as balloon aortic valvuloplasty. This limits our ability to analyse the relationship between disease patterns and outcomes across FIT and consultant cases and may introduce systemic bias in how these results should be interpreted. Finally, due to technical issues with linking the national PCI database to post-discharge outcomes, as seen in the original UK-BCIS CHIP Score study,^5^ we could not assess longer-term MACCE rates. Consequently, whether in-hospital outcome differences impact long-term results remains uncertain.

## Conclusion

The in-hospital outcomes of stable CHIP-PCI cases are comparable between supervised FIT and consultant primary operators. Training FIT as primary operators in CHIP-PCI appears to be feasible and safe and can be delivered within the standard training programme. The comparable outcomes may be driven by the two-operator ‘buddy’ effect that a FIT supervised by a consultant benefit from. Further research is warranted to better understand the impact of the FIT learning curve and to determine the optimal timing for safely initiating their journey into performing complex PCI procedures.

## supplementary material

10.1136/openhrt-2024-003131 online supplemental file 1

10.1136/openhrt-2024-003131 online supplemental file 2

## Data Availability

All data relevant to the study are included in the article or uploaded as supplementary information.
