# Supplementary material for: Complex high-risk indicated PCI (CHIP-PCI): is it safe to let fellows-in-training perform it as primary operators?
Source: Open Heart. 2025 Jan 30;12(1):e003131. doi: 10.1136/openhrt-2024-003131 (PMC11784205; doi:10.1136/openhrt-2024-003131)
Supplement: online supplemental file 1 [file openhrt-12-1-s001.pptx]

## Slide 1
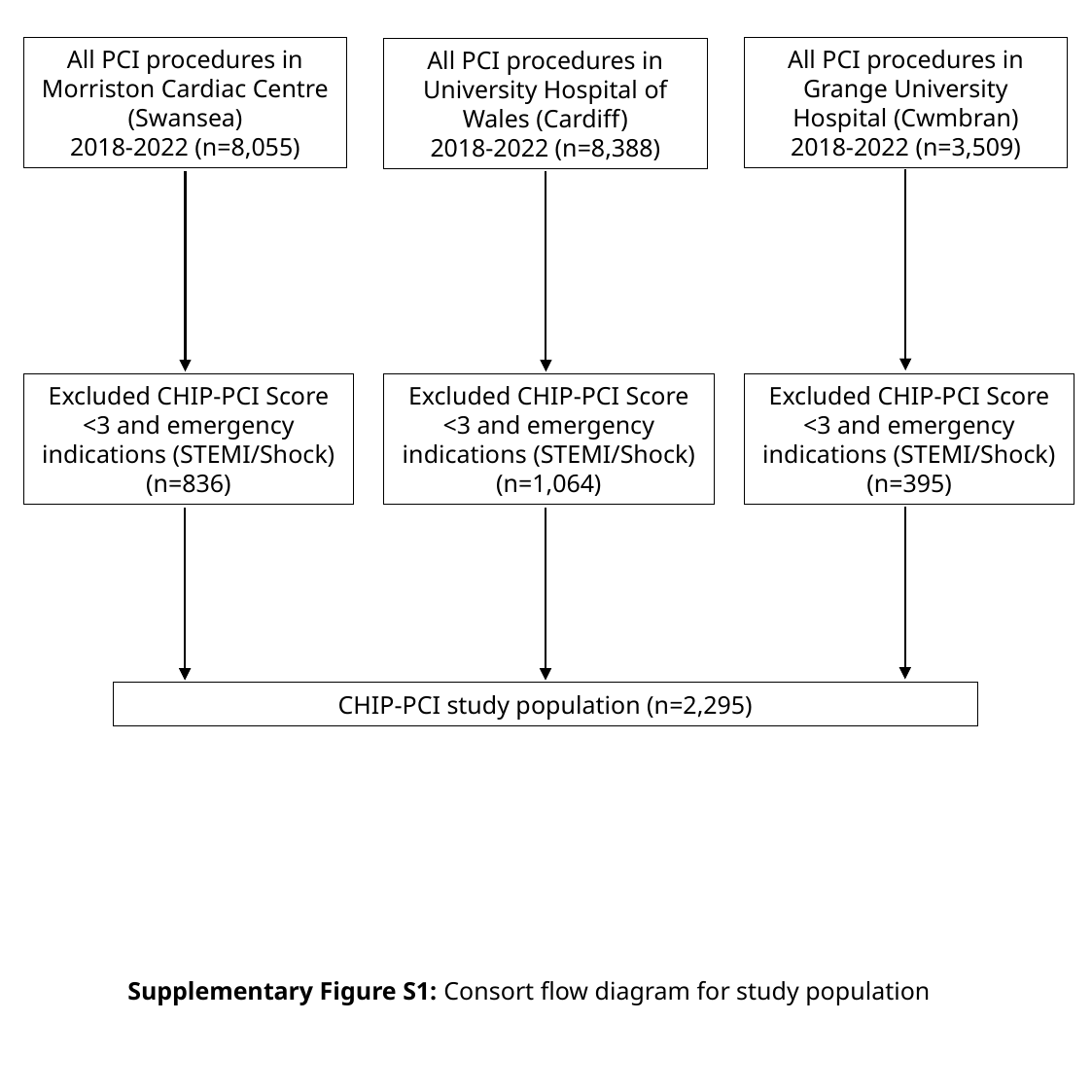

All PCI procedures in Grange University Hospital (Cwmbran)
2018-2022 (n=3,509)
All PCI procedures in Morriston Cardiac Centre (Swansea)
2018-2022 (n=8,055)
All PCI procedures in University Hospital of Wales (Cardiff)
2018-2022 (n=8,388)
Excluded CHIP-PCI Score <3 and emergency indications (STEMI/Shock)
(n=836)
Excluded CHIP-PCI Score <3 and emergency indications (STEMI/Shock)
(n=1,064)
Excluded CHIP-PCI Score <3 and emergency indications (STEMI/Shock)
(n=395)
CHIP-PCI study population (n=2,295)
Supplementary Figure S1: Consort flow diagram for study population
