# Supplementary material for: Complex high-risk indicated PCI (CHIP-PCI): is it safe to let fellows-in-training perform it as primary operators?
Source: Open Heart. 2025 Jan 30;12(1):e003131. doi: 10.1136/openhrt-2024-003131 (PMC11784205; doi:10.1136/openhrt-2024-003131)
Supplement: online supplemental file 2 [file openhrt-12-1-s002.docx]

| **Supplementary Table S1.** Variables with missing values included in the analysis and mitigated with multiple imputations with chained equations. Abbreviations as per main manuscript. | |
| --- | --- |
| **Variable** | **% Missing** |
| Age | 0 |
| CHIP Score | 0 |
| BMI | 11.8 |
| Age >80 | 0 |
| Female | 0.6 |
| ACS | 0 |
| CCS 3+ | 50.8 |
| NYHA 3+ | 82.7 |
| Diabetes | 1.6 |
| EF <30% | 2.9 |
| Trainee status | 0 |
| CHIP Score 4+ | 0 |
| CHIP Score 5+ | 0 |
| Smoking history | 55.5 |
| FH of CAD | 7.8 |
| Hypertension | 0 |
| Hypercholesterolaemia | 0 |
| Previous MI | 0 |
| Previous CABG | 0 |
| Previous PCI | 0 |
| PVD | 0 |
| Valve disease | 0 |
| Stroke | 0 |
| Renal disease | 3.9 |
| LMS PCI | 0 |
| 3-vessel PCI | 0 |
| Graft PCI | 0 |
| Intracoronary imaging | 0 |
| Pressure wire | 0.7 |
| Femoral access | 0 |
| Dual access | 0 |
| Length >60mm | 0 |
| Microcatheter | 1.2 |
| Cutting/Scoring | 1.2 |
| Rotational atherectomy | 1.2 |
| IVL | 1.2 |
| Mechanical LV support | 0 |
| Tamponade | 0 |
| Dissection | 0 |
| Shock induction | 0 |
| Perforation | 0 |
| Slowflow | 0 |
| DC cardioversion | 0 |
| Sidebranch loss | 0 |
| In-hospital death | 0 |
| In-hospital MACCE | 0 |
